# Malaria Burden and Associated Risk Factors in an Area of Pyrethroid-Resistant Vectors in Southern Benin

**DOI:** 10.4269/ajtmh.22-0190

**Published:** 2022-07-18

**Authors:** Manfred Accrombessi, Martin C. Akogbeto, Edouard Dangbenon, Hilaire Akpovi, Arthur Sovi, Boulais Yovogan, Constantin Adoha, Landry Assongba, Aurore Ogouyemi-Hounto, Germain Gil Padonou, Charles Thickstun, Mark Rowland, Corine Ngufor, Natacha Protopopoff, Jackie Cook

**Affiliations:** ^1^ Faculty of Infectious and Tropical Diseases, Disease Control Department, London School of Hygiene and Tropical Medicine, London, United Kingdom;; ^2^ Centre de Recherche Entomologique de Cotonou (CREC), Cotonou, Benin;; ^3^ UER Parasitology Mycology, Health Science Faculty, Abomey-Calavi University; National Malaria Control Program, Ministry of Health, Cotonou, Benin;; ^4^ School of Epidemiology and Public Health, Faculty of Medicine, University of Ottawa, Ottawa, Canada;; ^5^ Medical Research Council (MRC) International Statistics and Epidemiology Group, London School of Hygiene and Tropical Medicine, London, United Kingdom

## Abstract

Malaria remains the main cause of morbidity and mortality in Benin despite the scale-up of long-lasting insecticidal nets (LLINs), indoor residual spraying, and malaria case management. This study aimed to determine the malaria burden and its associated risk factors in a rural area of Benin characterized by high net coverage and pyrethroid-resistant mosquito vectors. A community-based cross-sectional survey was conducted in three districts in southern Benin. Approximately 4,320 randomly selected participants of all ages were tested for malaria using rapid diagnostic tests within 60 clusters. Risk factors for malaria infection were evaluated using mixed-effect logistic regression models. Despite high population net use (96%), malaria infection prevalence was 43.5% (cluster range: 15.1–72.7%). Children (58.7%) were more likely to be infected than adults (31.2%), with a higher malaria prevalence among older children (5–10 years: 69.1%; 10–15 years: 67.9%) compared with young children (< 5 years: 42.1%); however, young children were more likely to be symptomatic. High household density, low socioeconomic status, young age (< 15 years), poor net conditions, and low net usage during the previous week were significantly associated with malaria infection. Malaria prevalence remains high in this area of intense pyrethroid resistance despite high net use. New classes of LLINs effective against resistant vectors are therefore crucial to further reduce malaria in this area.

## INTRODUCTION

During the past few decades, long-lasting insecticidal nets (LLINs) have significantly contributed to the decline in malaria morbidity and mortality across sub-Saharan Africa (SSA).^1^ However, the progress achieved so far has stalled in many countries, with some seeing an increased burden in the last few years.^2^ COVID-19-related disruptions in the provision of malaria prevention, diagnosis, and treatment appear to have contributed to an increase in the number of cases in 2020.^3^ In Benin, malaria remains a serious public health issue despite the scale-up of LLINs, indoor residual spraying, and malaria case management.^4^^,^^5^ According to the national health statistics, 2,719,608 malaria cases were recorded in 2019 representing 45.5% of the overall outpatient diagnoses with 3,509 deaths attributable to malaria.^6^ In 2018, more than 90% of households declared to have at least one insecticide-treated net, and 71% had slept under nets the night before the survey.^7^

The emergence of insecticide resistance in malaria vectors, particularly to pyrethroids, raises concerns about the continued effectiveness of pyrethroid-only LLINs.^8^^–^^10^ In Benin, several studies have highlighted that a high proportion of malaria vectors are resistant to the pyrethroids used on insecticide-treated nets, including permethrin, deltamethrin, and alpha-cypermethrin,^11^^–^^15^ resulting in low mosquito mortality rates.^16^^,^^17^ However, the precise impact of resistance on malaria vector control efficacy is not fully known.^18^^–^^20^ In addition to insecticide resistance, a recent review has suggested that the resurgence of malaria cases may also be due to reductions in net usage and substandard nets.^21^

Due to concerns about the potential failure of current control tools, new types of LLINs have been developed to sustain malaria infection reduction.^22^^,^^23^ In preparation for a trial testing these new classes of LLINs, this study assessed the malaria infection burden and associated risk factors in southern Benin, a malaria-endemic setting with pyrethroid-resistant malaria vectors.

## MATERIALS AND METHODS

### Study area and design.

A community-based cross-sectional survey was conducted between October and November 2019 in three districts (Covè, Zagnanado, and Ouinhi) located in southern Benin. The survey was part of pre-intervention activities of a cluster randomized controlled trial designed to assess the impact of two novels dual-active ingredient LLINs (Royal Guard^®^ net combining alpha-cypermethrin and pyriproxyfen; and Interceptor^®^ G2 net incorporating alpha-cypermethrin and chlorfenapyr) for control of malaria transmitted by pyrethroid-resistant vectors, a part of the “New Nets Project”. The trial protocol has been fully described elsewhere.^24^

Malaria is highly endemic in the study area and malaria transmission occurs year-round. There are two rainy seasons, from April to July and from October to November. Malaria prevalence in the region was 36.5% in children under 5 years old according to the demographic health survey in 2018.^7^ The main vector control consists of the distribution of pyrethroid-only LLINs through universal coverage campaigns every 3 years (the most recent conducted in 2017) and routine service delivery to pregnant women during antenatal care and to children under 5 years during extended programs on immunization. An entomological survey conducted concurrently with this study,^25^ showed that *Anopheles coluzzii* and *Anopheles gambiae *s.s. were the main malaria vectors and both were highly resistant to pyrethroids mediated by multiple resistance mechanisms mainly L1014F *kdr* mutation (> 80%) and overexpressed mixed-function oxidases. The indoor and outdoor entomological inoculation rate (EIR) was 21.6 and 5.4 infected bites/person/month, respectively.^25^

### Study population.

A census was carried out in June 2019 in the 123 villages of the study area. Houses were numbered, georeferenced, and information on the household’s residents (name, sex, age, number of sleeping areas and LLINs, relationship with the household head) were collected. Sixty clusters were identified with each cluster comprising of one village or a group of villages for an average of 200 households (approximately 1,200 residents). All resident adults or children over 6 months of age willing to participate and providing consent (or guardian’s consent) were eligible for the study.

### Study procedures.

To achieve adequate participant enrolment, enhanced community sensitization activities were conducted before and during the survey with the support of hamlet leaders and community health workers. For malaria infection prevalence assessment, 72 individuals per cluster were randomly selected (total of 4,320 participants), stratified by age (< 5 years, 5–9 years, 10–14 years, ≥ 15 years) from each of the 60 clusters.

The survey included two components: 1) a household survey and 2) a clinical survey. During the household survey, a questionnaire was administered to obtain information on possible risk factors for malaria and sampled persons were tested for malaria using rapid diagnostic tests (Malaria Pf HRP2 Ag RDT, Carestart^®^).

#### Household survey.

Age-stratified individuals were randomly selected using two-stage cluster sampling from the census list generated during the registration activity. A maximum of two members was selected per house. The household questionnaire included information on gender, age, educational status and occupation, socioeconomic status, vector control measures used (number of nets, type and condition of nets, duration of usage), previous malaria cases, and care-seeking behaviors. The physical conditions of nets was assessed visually and categorized as good, average, and poor condition based on consensus between two field staff. We also assessed the presence of potential breeding sites such as water taps, open gutters, semi-open gutters, wells, puddles, septic tanks, or rivers in the proximity of the household.

#### Malaria testing.

Participants were tested with an rapid diagnostic test (RDT), regardless of symptoms. A temperature record was taken for all participants using an infrared thermometer. Symptomatic malaria infection was defined by a positive RDT plus fever (temperature ≥ 37.5°C) or a history of fever in the last 48 hours. Treatment was provided free of charge if they were found positive. Any participants with severe malaria or any diagnosed ailments that could not be treated by study staff were referred to the nearest health facilities.

Hemoglobin levels were measured with a Hemocue device in children aged 5 years old and under. Anemia was defined as a hemoglobin concentration below 110 g/L. Severe, moderate, and mild anemia were respectively defined as hemoglobin levels less than 70 g/L, between 70–99 g/L and 100–109 g/L.^26^

Long-lasting insecticidal net coverage was assessed with the following indicators^27^^,^^28^: 1) *Household net ownership* defined as the proportion of households with at least one bed net; 2) *Household net access* which is the proportion of households with enough nets for every two family members (based on one net for every two members); 3) *Population net access* corresponding to the proportion of individuals with access to bed nets within the households (assuming that each bed net in a household can be used by two people), and 4) *Population net use* representing the proportion of individuals who reported sleeping under a bed net the previous night. Net usage was also assessed during the previous week (net used 0–4 nights, i.e., few nights, 5–6 nights, i.e., most nights, 7 nights, i.e., all nights). The physical quality of the nets were also broadly assessed by eye, with field workers classifying nets as good, average, or poor, based on its condition.

Mosquito collections were undertaken across all villages, between September and October 2019. In each village, four houses were selected for mosquito sampling using human landing catches (HLCs). To ensure the supervision of mosquito collectors, the first house was randomly selected from the study census list, while the other three were chosen within a radius of 15–20 m around the first one. Four collectors were required per house (one indoor and one outdoor replaced by two others after 6 hours of collection). Details on entomological data collection as well as baseline characteristics have been fully detailed elsewhere.^25^

### Data management and statistical analysis.

All data collected during the survey were recorded in electronic forms on smartphones installed with Open Data Kit (ODK) collect and analyzed with STATA version 16 (Stata Corp LP, College Station, TX).

Descriptive statistics and 95% confidence intervals were used to summarize demographic data. Mixed-effect logistic regression models were used to assess risk factors with clusters included as a random effect. Variables with *P* values below 0.2 were included in multivariate analyses and were eliminated step by step using the backward selection procedure. Only variables whose *P* < 0.05 were retained in the final model. For variables with more than two categories, a *P* value of the global test is given. Spatial similarities in malaria prevalence and EIR were examined visually using QGIS, with “natural breaks” to display the most variation in the data, given the histogram for each variable. A linear regression was used to assess the association between cluster-level malaria infection prevalence and cluster-level entomological indicators (mosquito density, EIR).

Risk factors assessed included characteristics at the household level (number of residents, head of the household gender and education, socioeconomic status, ethnic group, net ownership, and access), and individual level (age, gender, net usage). Household socioeconomic status was created by using principal component analysis with the following variables included: type of lighting, access to water, type of roof, type of floor, type of toilet, household head level of education, household crowding, and ownership of assets (motorbike, television, bike, radio, sheep, bed, phone).

### Ethical statement.

The study protocol was approved by the Benin Ministry of Health ethics committee (Reference N°6/30/MS/DC/SGM/DRFMT/CNERS/SA) and by the institutional ethics review board of the London School of Hygiene and Tropical Medicine (N°16237). All participation was voluntary. Written informed consent was obtained from an adult guardian in the household or given by the participant if over 18 years. Assent was sought for children over 10 years.

## RESULTS

A total of 216,138 inhabitants in 54,143 households were identified as part of the census. In the baseline survey, 4,441 randomly selected participants from 3,027 households were included.

### Sociodemographic characteristics of the study population.

The mean number of people living in households was 5.3 (standard deviation [SD] ± 2.6) inhabitants. The number of sleeping spaces in the household was on average 2.7 (SD ± 1.3). The majority of heads of household had no formal education (71.4%) and the main ethnic group was “Fon” (77.2%). Mosquito breeding sites were observed close to 76.9% of households. Females represented 50.8% of the participants; children under 5 years old, 5–10 years, 10–15 years, and above to 15 years accounted for 16.5%, 14.4%, 13.6%, and 55.4%, respectively. Demographic, socioeconomic, and household characteristics of study participants are detailed in Table 1.

**Table 1:** Baseline characteristics of the study population

Characteristics		Proportion or mean
**Cluster-level**		
Number of clusters		60
Number of households per cluster	Mean (range)	904 (82–5403)
Proportion of children under 10 years per cluster, %	Mean (range)	30.9 (25–35.2)
Cluster with high socioeconomic status, % (n)	Yes	62.7 (37)
**Household-level**		
Number of households enrolled		3,027
People living in the household	Mean (± SD)	5.3 (±2.6)
Number of sleeping spaces	Mean (± SD)	2.7 (±1.3)
Head of household gender, % (n)	Male	83.4 (2,525)
	Female	16.6 (502)
Head of household education level, % (n)	No education	71.4 (2,160)
	Primary education	17.2 (521)
	Lower secondary education	7.3 (220)
	Higher secondary education	2.7 (81)
	College/University	1.4 (45)
Ethnic group, % (n)	Fon	77.2 (2,336)
	Other*	22.8 (691)
Presence of potential breeding sites, % (n)	Yes	76.9 (2,209)
**Individual-level**		
Number of participants enrolled		4,441
Participant gender, % (n)	Male	49.1 (2,182)
	Female	50.8 (2,259)
Age, years % (n)	≤ 5	16.5 (735)
	5–10	14.4 (638)
	10–15	13.6 (606)
	> 15	55.4 (2,462)
Education level, % (n)	Not in the age of education	16.5 (735)
	Not educated	52.5 (2,333)
	Educated	30.9 (1,373)

SD = standard deviation.

* Other ethnic groups: Mahi, Holli, Yoruba, Goun.

### Household LLIN characteristics.

Table 2 presents the characteristics of bed nets recorded in the households. The number of bed nets in the household was on average 2.5 (SD ± 1.3). Most of the nets found were insecticide-treated nets (96.5%). The majority of the bed nets were provided during the previous national campaign of 2017 (91%) with others supplied through public health routine activities (8%, from antenatal care visits and expanded program immunization). PermaNet^®^ 2.0 was the most common LLIN type observed in the households (64.3%). Most bed nets had been used for more than 2 years (92.7%). The proportion of LLIN in poor condition was 37.9%. Household net ownership, household net access, population net access, and population net use were 95.8%, 55.9%, 79.5%, 95.8%, respectively. The majority of the participants (88.4%) declared to have slept under an LLIN every night during the previous week. Children under 5 years old had slept more often under a net every night the previous week in comparison to others (< 5 years: 93.1%; 5–10 years: 88%; 10–15 years: 84.9%; > 15 years: 87.3%).

**Table 2:** Characteristics of long-lasting insecticidal nets presented in the household

Characteristics		Proportion or mean
**Household-level**		
Number of households enrolled		3,027
Household net ownership, % (n)	Yes	95.8 (2,902)
Household net access, % (n)	Yes	55.9 (1,691)
Population net access, % (95% CI)	Yes	79.5 (78.9–80.1)
Number of nets (any type) in the household	Mean (±SD)	2.5 (±1.3)
Number of LLIN in the household	Mean (±SD)	2.3 (±1.3)
**Individual-level**		
Number of participants enrolled		4,441
Number of participants enrolled with a net		4,088
Number of participants enrolled with an LLIN		3,949
Origin of LLIN, % (n)	National campaign (2017)	91.0 (3,595)
	Healthcare facility (ANC)	6.4 (251)
	Healthcare facility (EPI)	1.6 (65)
	Public market	0.4 (16)
	Other	0.6 (22)
Proportion of LLIN observed, % (n)	Yes	99.0 (3,910)
Type of LLIN, % (n)	PermaNet 2.0	64.3 (2,489)
	Dawa plus	9.6 (374)
	Olyset Net	7.0 (271)
	Duranet	1.4 (53)
	Yorkool	0.5 (21)
	Other*	5.7 (221)
	Unknown	11.4 (443)
Duration usage of LLIN, % (n)	≤ 1 year	7.2 (286)
	2 years	64.3 (2,540)
	3 years	21.1 (833)
	> 3 years	7.3 (290)
LLIN conditions, % (n)	Good	18.9 (732)
	Average	43.2 (1,671)
	Poor	37.9 (1,468)
Population LLIN use, % (n)	Yes	95.8 (3,782)
Net usage the week before the visit, % (n)	All nights	88.4 (3,490)
	Most of the nights	8,6 (338)
	Few nights	3.1 (121)

ANC = antenatal care visit; EPI = expended program immunization; LLIN = long-lasting insecticidal net; SD = standard deviation.

* Most important other types of bed nets observed were impregnated by deltamethrin (88.2%, 195/221).

### Prevalence of malaria and analysis of potential risk factors.

The prevalence of malaria infection was 43.5% (cluster range: 15.1–72.7%) (Table 3). Children less than 5 years old (malaria prevalence 42.1%), aged between 5–10 years (69.1%), and 10–15 years (67.9%) were most likely to be infected with malaria in comparison to adult participants (31.2%). However, among infected participants, the proportion of symptomatic cases was higher among children under 5 years (33.3%) in comparison to older children (5–10 years: 28.9%; 10–15 years: 23.7%) and adults (19.2%). The proportion of people presenting a fever or history of fever in the last 48 hours was 18.7% (829/4,441) of which 57.2% were malaria positive. Among children under 5 years old, anemia was present in 75.9% (cluster range: 33.3–100%).

**Table 3:** Malaria indicators in the study population

Characteristics		Proportion % (n)
Number of participants enrolled, n		4,441
History of fever in the past 48 hours	Yes	12.6 (562)
Fever* at the time of visit	Yes	8.0 (356)
Hemoglobin test performed among children under 5 years	Yes	99.5 (731)
Anemia† among children under 5 years old	Yes	75.9 (555)
Malaria rapid diagnostic test performed	Yes	99.7 (4,428)
Malaria infection	Yes	43.5 (1,924)
Malaria infection by age category	≤ 5 years	42.1 (309)
	5–10 years	69.1 (440)
	10–15 years	67.9 (410)
	> 15 years	31.2 (765)
Proportion of symptomatic cases‡ among infected per age	≤ 5 years	33.3 (103/309)
	5–10 years	28.9 (127/440)
	10–15 years	23.7 (97/410)
	> 15 years	19.2 (147/765)
Proportion of malaria among participants with fever or history of fever in the past 48 hours	Yes	57.3 (474)

* Fever: infrared frontal temperature ≥ 37.5°C.

† Hemoglobin (Hb) < 110 g/L.

‡ Symptomatic cases: defined by the presence of malaria infection without fever or history of fever in the last 48 hours.

Household population density, socioeconomic status, gender, ethnic group, age, households with enough LLIN, net use the previous week, and net usage duration were factors associated with malaria infection (Table 4). After adjusting for confounding factors, the risk of malaria infection was significantly higher with an increasing number of people living in the house (adjusted odds ratio [aOR] 1.32, 95% CI 1.05–1.69 for households having between three and seven members; and aOR 1.54, 95% CI 1.16–2.04 for households having more than three members in comparison to households with less than three members). People living in households with average (aOR1.43, 95%CI 1.19–1.69) and low (aOR 1.44, 95%CI 1.16–1.79) socioeconomic levels were more at risk of malaria infection than those living in households with high socioeconomic status. The age groups at highest risk were children under 5 years (aOR 1.66, 95% CI 1.38–1.99), between 5 and 10 years (aOR 5.31, 95% CI 4.33–6.53) and 10–15 years (aOR 4.88, 95% CI 3.96–6.03) in comparison to participants over 15 years. Participants who reported sleeping under nets a few nights (< 4 nights) the previous week were also more likely to be infected (aOR 1.95, 95% CI 1.29–2.94) in comparison to those who slept under nets every night. Also, household members using nets in poor condition were more likely to be infected (aOR 1.34, 95% CI 1.07–1.74) in comparison to those owning nets in good condition.

**Table 4:** Risk factors associated with malaria infection in an area of pyrethroid-resistant vectors in Benin

Factors		Malaria positive % (n)	Univariate analysis		Multivariate analysis	
			OR* (95% CI)	*P* value	aOR† (95% CI)	*P* value
**Household-level**						
People living in the household	< 3	29.2% (148)	1		1	
	3–7	43.8% (1,382)	1.82 (1.47–2.24)	< 0.001	1.29 (1.01–1.65)	0.03
	> 7	51.6% (394)	2.36 (1.84–3.02)		1.47 (1.10–1.96)	
Household socioeconomic status	High	36.7% (542)	1		1	
	Average	46.8% (685)	1.32 (1.12–1.57)	0.002	1.40 (1.12–1.75)	< 0.001
	Low	46.8% (697)	1.33 (1.10–1.61)		1.41 (1.17–1.72)	
Household net access	No	40.4% (989)	1			
	Yes	47.2% (935)	1.23 (1.08–1.39)	0.002		
**Individual-level**						
Gender	Female	41.2% (928)	1			
	Male	45.7% (996)	1.18 (1.05–1.34)	0.007		
Ethnic group	Other	39.4% (408)	1			
	Fon	44.7% (1,516)	1.17 (0.95–1.45)	0.13		
Participant age	> 15 years	31.2% (765)	1		1	
	10–15 years	67.9% (410)	5.15 (4.23–6.28)		4.96 (4.01–6.13)	
	5–10 years	69.1% (440)	5.43 (4.47–6.61)	< 0.001	5.52 (4.48–6.81)	< 0.001
	≤ 5 years	42.1% (309)	1.66 (1.39–1.98)		1.68 (1.39–2.03)	
Duration usage of LLIN	≤ 1 year	37.4% (116)	1			
	2 years	43.7% (1,139)	1.41 (1.07–1.84)			
	3 years	47.2% (408)	1.58 (1.17–2.13)	0.01		
	> 3 years	37.0% (110)	1.15 (0.78–1.70)			
Net usage the previous week	All nights	43.2% (1,552)	1		1	
	Most of nights	43.3% (154)	1.18 (0.91–1.54)	0.04	1.01 (0.76–1.36)	0.02
	Few nights	51.5% (67)	1.60 (1.09–2.35)		1.83 (1.19–2.83)	
Net conditions	Good	38.4% (289)	1		1	
	Average	42.9% (732)	1.23 (0.99–1.53)	0.002	1.23 (0.97–1.55)	0.04
	Poor	46.1% (705)	1.48 (1.17–1.85)		1.37 (1.07–1.74)	

aOR = adjusted odds ratio; LLIN = long-lasting insecticidal nets; OR = odds ratio.

* Adjusted for within-cluster effect.

† Adjusted for significant variables associated with malaria infection and within-cluster effect.

**Figure 1.**
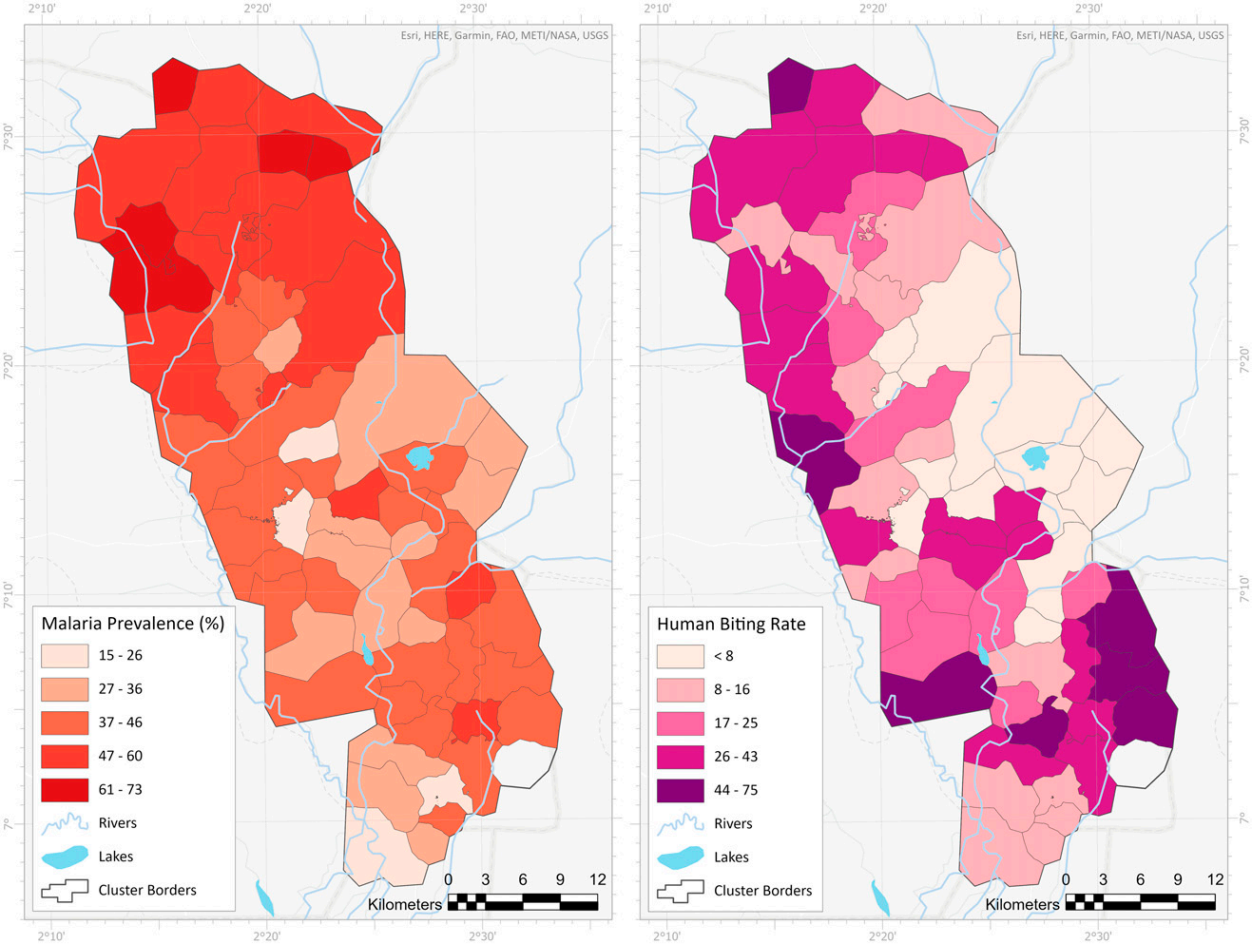
Comparison map of malaria prevalence and vector density within the 60 study clusters. This figure appears in color at www.ajtmh.org.

We observed a similar spatial distribution of malaria vector density and the prevalence of infection within clusters (Figure 1), with both indicators being high in the north of the study area. At the cluster level, increased malaria prevalence was associated with higher human biting rate and EIR (Supplemental Figure 1, Supplemental Table 1) although R squared values were very low suggesting not a strong relationship.

## DISCUSSION

This study examined risk factors for malaria infection in an area of intense transmission and the presence of pyrethroid-resistant vector mosquitoes.^15^^,^^16^ There is limited cross-sectional data available for this part of Benin and the current study represents a detailed analysis of the epidemiological picture, and compares it to the entomological outcomes reported in the same area.

Our data showed a high prevalence of malaria infection (43.5%) despite the high household net ownership and population net use. These findings are consistent with previous studies conducted in Benin.^29^^–^^31^ Despite there being several years since the last universal net campaign, net ownership, access, and use remained very high. The concerningly high prevalence, despite high net use, may be partially explained by the high level of pyrethroid resistance in the local malaria vectors.^21^^,^^28^ Indeed, entomological surveys conducted simultaneously to this study have confirmed the scale of pyrethroid resistance with mosquitoes mortality rates at 24, 48, and 72 hours postexposure lower than 40% for alpha-cypermethrin and permethrin insecticides and a frequency of the L1014F *kdr* mutation over 80%.^25^ Several studies have also highlighted the increasing role of pyrethroid-resistant *Anopheles* vectors in malaria transmission.^9^^,^^12^^,^^14^^,^^32^ However, this observation might be also related to the age and condition of the nets.^33^ In fact, we observed in this study that individuals using nets in poor condition were 1.37-fold more likely to be infected than those sleeping under nets in good condition. As the condition of the nets were only assessed by eye, rather than using the WHO hole assessment criteria, this finding should not be overinterpreted.

Children under 15 years were most likely to be infected with malaria, with children aged between 5 and 15 years at higher risk than those under 5 years. Traditionally, children aged under 5 years have been thought to be at the highest risk of malaria infection. Changes in infection burden by age group have also been reported elsewhere in SSA.^34^^–^^36^ This has important implications for targeting interventions and surveillance in SSA. Several studies have previously suggested that reduced transmission might lead to increased susceptibility to malaria infection among older children due to lower acquired immunity.^37^^–^^39^ However, good malaria case management in children under 5 years old might also partially explain the shift in infection burden. Despite infection being higher in older children, the most severe health consequences continue to mainly affect the youngest children.^40^^,^^41^ Indeed, 33.3% of the youngest children (< 5 years) tested for malaria had fever whereas 28.9%, 23.7%, and 19.2% of infected children between 5–10 years, 10–15 years, and adults (> 15 years) presented malaria related-morbidity, respectively. Furthermore, even though more asymptomatic, older children and adults are important drivers of malaria infection as they might not seek treatment and remain a reservoir for infection, undermining elimination efforts.^42^^–^^44^ They should, therefore, be considered for malaria public health interventions.

Socioeconomic status was also a factor significantly associated with malaria infection. Indeed, a recent systematic review has shown that an improved socioeconomic status would reduce dramatically the burden of malaria in SSA.^45^ Moreover, participants not sleeping under nets usually were more likely to be infected. This is in line with other studies highlighting the importance of population net usage despite the access.^21^^,^^46^

We observed a similar spatial distribution of malaria infection and malaria vector density and EIR over the clusters, highlighting the usefulness of both indicators, as well as a loose association between increasing malaria prevalence and increasing mean human biting rate and EIR.^47^^,^^48^ Throughout the trial, we will assess the relationship between the indicators over season to see how each net type affects them.

There is little information in Benin regarding the effect of increasing LLIN use and increasing insecticide resistance on malaria vector control and impact on malaria infection.^19^^,^^20^^,^^49^ These baseline results suggested that pyrethroid LLIN provide, at best, only partial protection against malaria and the importance of trialing novel LLIN technologies and approaches.^20^^,^^50^ The development and impact of insecticide resistance on malaria outcomes are key indicators to monitor.^17^^,^^20^^,^^25^^,^^51^^,^^52^

Long-lasting insecticidal nets remain a cornerstone of malaria control in Africa; however, the progress achieved so far is seriously threatened by pyrethroid-resistant malaria vectors. The increasing insecticide resistance in malaria vectors could have dramatic implications for malaria control and requires urgent remedial actions.^9^ It is, therefore, crucial to assess new classes of LLINs effective against resistant vectors to sustaining malaria reduction. Piperonyl butoxide-treated insecticidal nets have recently proven their effectiveness in malaria reduction in East Africa, though evidence of their epidemiological impact in West Africa is lacking.^53^^,^^54^ Other such novel generations of ITNs (pyrethroid-pyriproxyfen and pyrethroid-chlorfenapyr ITNs) are currently under evaluation in communities in Benin as part of the “New Nets Project, which aims to accelerate the impact of these new technologies across SSA.^22^^,^^23^

## Supplemental files


Supplemental materials
