# Supplementary material for: Malaria Burden and Associated Risk Factors in an Area of Pyrethroid-Resistant Vectors in Southern Benin
Source: Am J Trop Med Hyg. 2022 Jul 18;107(3):681–8. doi: 10.4269/ajtmh.22-0190 (PMC9490648; doi:10.4269/ajtmh.22-0190)
Supplement: Supplementary file 1 [file tpmd220190.SD1.pdf]

Supplemental Table S1. Relationship between malaria prevalence and entomological indicators at cluster level (linear regression)

Entomological indicators	Mean (95% CI)	Cluster malaria prevalence			
		Coefficient	95% CI	p-value	R2
Indoor human biting rate (b/p/n)	27.92 (26.59-29.28)	0.17	0.03-0.32	0.01	0.09
Indoor entomological inoculation rate (ib/p/m)	21.97 (20.79-23.18)	0.16	0.07-25.5	0.001	0.17
Outdoor human biting rate (b/p/n)	19.12 (18.02-20.26)	0.24	0.04-0.45	0.02	0.08
Outdoor entomological inoculation rate (ib/p/m)	6.02 (5.41-6.67)	0.12	-0.19-0.42	0.45	0.01

Abbreviations
CI: Confidence interval
b/p/n: bite/person/night
ib/p/m: infected bites/person/month

Supplemental Figure S1. Scatter plot comparing the relationship between malaria prevalence rates and entomological indicators at cluster level.

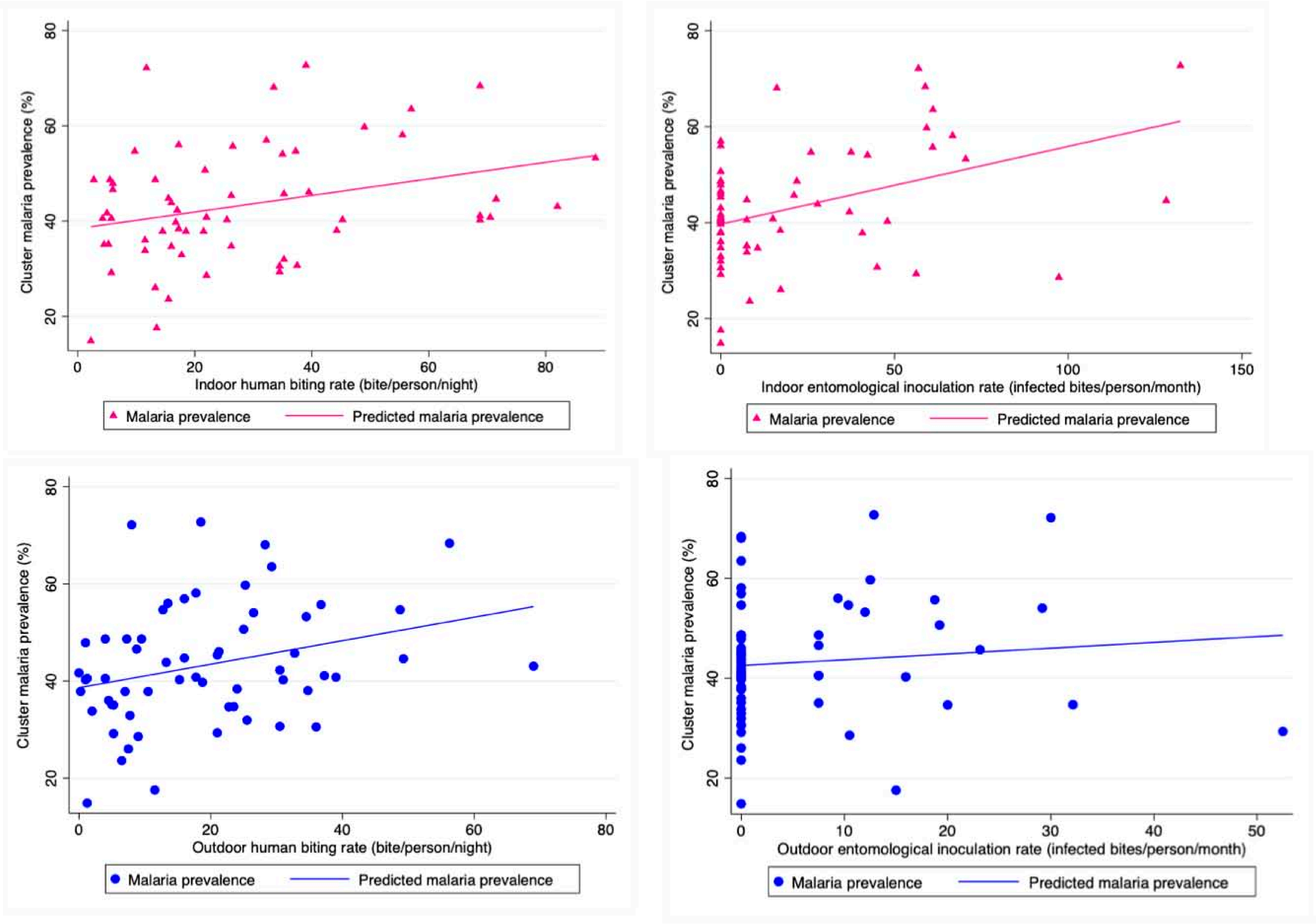

Cluster malaria prevalence (%)
80
60
40
20
0
20
40
60
80
Indoor human biting rate (bite/person/night)
Malaria prevalence
Predicted malaria prevalence
Cluster malaria prevalence (%)
80
60
40
20
0
50
100
150
Indoor entomological inoculation rate (infected bites/person/month)
Malaria prevalence
Predicted malaria prevalence
Cluster malaria prevalence (%)
80
60
40
20
0
20
40
60
80
Outdoor human biting rate (bite/person/night)
Malaria prevalence
Predicted malaria prevalence
Cluster malaria prevalence (%)
80
60
40
20
0
10
20
30
40
50
Outdoor entomological inoculation rate (infected bites/person/month)
Malaria prevalence
Predicted malaria prevalence
